# Insecticide resistance and the role of target-site insensitivity mutations among malaria vectors in China: A systematic review and meta-analysis

**DOI:** 10.1186/s13071-025-07020-6

**Published:** 2025-09-24

**Authors:** Zhiquan He, Dan Wang, Yuanjing Kou, Ying Liu, Dongyang Zhao, Chengyun Yang, Ruimin Zhou, Hongwei Zhang, Yan Deng

**Affiliations:** 1 Department of Parasite Disease Control and Prevention, Henan Center for Disease Control and Prevention, Zhengzhou, China; 2 Henan Key Laboratory of Pathogenic Microorganisms, Zhengzhou, China; 3 Henan Provincial Medical Key Laboratory of Parasitic Pathogen and Vector, No. 105 South Agricultural Road Zhengdong New District, Zhengzhou, 450016 China

**Keywords:** Malaria, Insecticide resistance, *Anopheles*, Knockdown resistance (*kdr*), Acetylcholinesterase-1 (*ace-1*), Meta-analysis, China

## Abstract

**Background:**

Malaria, which is transmitted by *Anopheles* mosquitoes (Diptera: Culicidae), remains a critical global public health problem. Vector control interventions, particularly insecticide-based strategies, are pivotal for malaria control and elimination, as the efficacy of these interventions is heavily dependent on the high susceptibility of *Anopheles* mosquitoes to insecticides. However, insecticide resistance in mosquito vectors poses a considerable threat to the sustainability of these control efforts. Notably, no synthesis data on insecticide resistance have been reported in China in recent decades.

**Methods:**

This systematic review and meta-analysis aimed to assess the mortality rates and frequency of knockdown resistance (*kdr*) and acetylcholinesterase-1 (*ace-1*) mutations in *Anopheles* mosquitoes. The Chinese National Knowledge Infrastructure, Wan Fang, PubMed, Embase, and Web of Science databases were searched from 2000 to 2024 to identify relevant articles. Meta-analysis was performed using R and Stata software.

**Results:**

Thirty articles reporting 30,065 *An. sinensis* were included. The pooled mortality rate for insecticide resistance was 61% [95% confidence interval (CI): 53–68]. The mortality rates of various insecticides were as follows: dichlorodiphenyltrichloroethane (DDT), 49%; deltamethrin, 47%; malathion, 81%; propoxur, 69%; permethrin, 61%; beta-cyfluthrin, 28%; fenitrothion, 82%; beta-cypermethrin, 48%; cyfluthrin, 59%; and lambda-cyhalothrin, 56%. Moreover, the frequency of knockdown resistance (*kdr*) to insecticides was 36%, whereas the frequency of acetylcholinesterase-1 (*ace-1*) resistance was 78%. *Kdr* genotype analysis revealed that 13% of the reported mosquitoes were homozygote resistant, 13% were heterozygote resistant, and 74% were zygote susceptible. *Ace-1* genotype analysis revealed that 42% of the reported mosquitoes were homozygote resistant, 25% were heterozygote resistant, and 33% were zygote susceptible.

**Conclusions:**

Of *An. sinensis*, 36% had *kdr* mutations, and 78% had *ace-1* mutations. These vectors were resistant to pyrethroid, organochlorine, carbamate, and organophosphate insecticides. To prevent the development of resistance to alternative insecticides, it is critical to target *Anopheles* mosquitoes with novel chemical insecticides or biocontrol approaches.

**Graphical Abstract:**

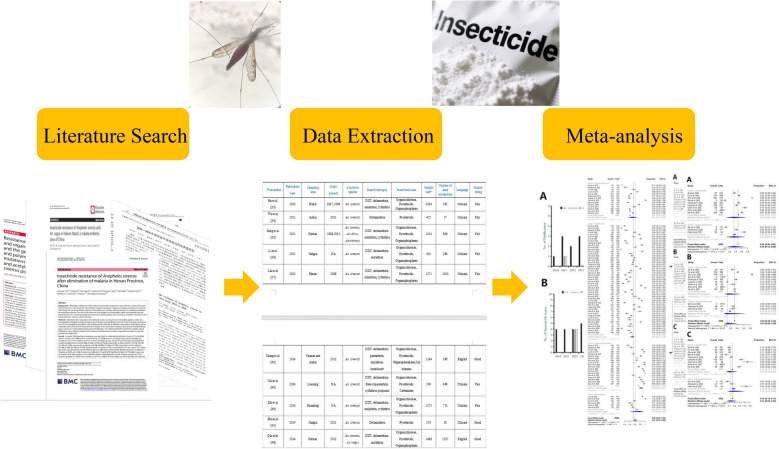

**Supplementary Information:**

The online version contains supplementary material available at 10.1186/s13071-025-07020-6.

## Background

Mosquito vectors can transmit a wide range of pathogens, including arboviruses, protozoan parasites, bacterial agents, and filariae nematodes, all of which collectively pose significant global public health challenges [1, 2]. Mosquitoes belong to three main genera: *Anopheles*, *Aedes*, and *Culex*. Malaria, which is transmitted by *Anopheles* mosquitoes, is one of the most significant life-threatening infectious diseases to humans worldwide [3]. According to the World Health Organization (WHO; 2024), the global number of malaria-related deaths reached 597,000, and there were approximately 263 million cases of malaria in 2023. The percentage of total malaria deaths among children aged under 5 years declined between 2000 and 2023, from 86.7% to 73.7% [4]. Vector control serves as a critical component of integrated malaria prevention and control strategies, particularly through interventions such as insecticide-treated bed nets (ITNs) and indoor residual sprayings (IRSs) [5]. However, insecticide resistance among most mosquito vector species poses a significant threat to the sustainability of conventional vector control strategies [6].

Insecticides are widely used worldwide because of their broad-spectrum, efficient, and long-lasting residual effects [7]. Six major classes of insecticides are approved for vector control, including the pyrethroids, organochlorines, carbamates, neonicotinoids, pyrroles, and organophosphates [4, 8–10]. The mechanisms underlying the widespread emergence of insecticide resistance have been well characterized. There are two main mechanisms of insecticide resistance: resistance mediated by target-site modifications and resistance resulting from increased metabolic detoxification rates [10]. Metabolic resistance occurs when increased activity of detoxifying enzymes accelerates the metabolism of insecticides, leading to their degradation before they reach the target site. The enzymes primarily involved in metabolic resistance include esterases (*ESTs*), glutathione S-transferases (*GSTs*), and the cytochrome P450 monooxygenase system (*P450s*) [11]. Previous studies revealed that, in *An. funestus*, a primary malaria vector in Central Africa, a 6.5-kb intergenic structural variation heightened the fitness costs of *CYP6P9a/b*-mediated resistance. The co-occurring *L119F-GSTe2* resistance further intensified disease transmission intensity across key endemic foci, including Cameroon [12, 13]. Target-site resistance arises from genetic mutations that structurally modify the insecticide’s intended protein targets, primarily the *sodium ion channels (SCs)*, acetylcholinesterase (AchE), and *γ-aminobutyric (GABA)* receptor–chloride channel complexes, which are critical for neural signaling in mosquito vectors [14–17]. The widespread phenomenon of knockdown resistance (*kdr*) in insects stems from mutations within *voltage-gated sodium channel* genes, which confer target-site insensitivity through structural alterations of insecticide-binding domains [18]. Mosquitoes possess two types of AChE enzymes, AChE1 and AChE2, which are encoded by the acetylcholinesterase-1 (*ace-1*) and *ace-2* genes, respectively. Only AChE1 is associated with insecticide resistance [19]. Despite achieving WHO malaria-free certification on 30 June 2021, China faces ongoing challenges from persistent *Anopheles* vector populations in historically endemic zones and the sustained importation of thousands of malaria cases annually, thus necessitating vigilant surveillance to sustain elimination status [20–22]. Historically, research in China has focused primarily on monitoring insecticide resistance in *Anopheles* mosquitoes across different provinces [23–25]. Based on previous surveillance data, this systematic review and meta-analysis aimed to calculate insecticide-related mortality rates and determine the frequencies of *kdr* and *ace-1* resistance mutations in China. These findings will support malaria vector control strategies, guide insecticide selection, and ultimately reduce the incidence of malaria.

## Methods

This systematic review was conducted in accordance with the Cochrane Collaboration and Preferred Reporting Items for Systematic Reviews and Meta-analyses (PRISMA) guidelines. It was also registered in the International Prospective Register of Systematic Review (PROSPERO) with the code CRD42025631834. The PRISMA checklist is provided in the Supporting Information (Additional file 1 Table S1).

### Search strategy

The Chinese National Knowledge Infrastructure (CNKI), Wan Fang, Web of Science, Embase, and PubMed electronic databases were searched from January 2000 to December 2024 to identify relevant articles published in English or Chinese. The search strategy was as follows: (“*Anopheles*” OR “Malaria vector” AND “Insecticide” AND “Resistance”). The reference lists of the included articles were also searched to identify additional eligible studies.

### Inclusion and exclusion criteria

Initially, two authors independently screened the titles and abstracts of the retrieved studies. The authors subsequently screened the full texts of the potentially eligible articles. The inclusion criteria were as follows: (i) English- or Chinese-language articles that reported *Anopheles* and (ii) articles in which the frequency of insecticide resistance or mortality rates related to insecticide exposure was reported or estimated. Studies that investigated *kdr* or *ace-1* mutation resistance were eligible for inclusion. The exclusion criteria were as follows: studies examining mosquitoes other than *Anopheles*; articles about malaria that did not mention insecticides; reviews; duplicated publications; overlapping datasets; and articles from which no data could be extracted.

### Data extraction and quality assessment

Two authors independently extracted the data. Disagreements were resolved through discussion or by consulting a third author. The following data were extracted from the included articles: first author, publication year, sampling area, study period, sample size, *Anopheles* species, insecticide type, insecticide concentration, number of dead mosquitoes, and *kdr*- or *ace-1*-resistance frequency. Additionally, if no reliable data were available, we input “NA” (not available) during extraction. The quality of the articles was measured using the Strengthening the Reporting of Observational Studies in Epidemiology (STROBE) checklist. Each section of the checklist contains subgroups, with points allocated according to their significance. The maximum score that can be obtained is 33, and the scores can be categorized as good, fair, or poor [26, 27].

### Statistical analysis

Resistance monitoring of malaria vectors was conducted in line with WHO guidelines. Susceptibility tests were carried out following the WHO guidelines for monitoring resistance in malaria vectors. Mosquito resistance status was interpreted in accordance with WHO guidelines, including confirmed resistance (CR) if mortality was < 90%, possible resistance (PR) if mortality was between 90% and 97%, and susceptible (S) if mortality was ≥ 98% [10]. Data analysis was carried out using R software (version 4.0.0) and STATA version 12.0 (STATA Corporation, College Station, Texas, USA) [28, 29]. The event rates and proportions [with confidence intervals (CI)] were calculated using the R software package [28]. The Cochran *Q* and *I*^*2*^ statistics were utilized to evaluate heterogeneity across the studies [30]. A Cochran *Q* test with *p* < 0.05 indicated significant heterogeneity. When the *I*^*2*^ value was greater than 75%, a random effects model was applied for pooled analysis. Sensitivity analysis was conducted using the leave-one-out method to assess each study’s impact on the overall results, and publication bias was evaluated using Begg’s or Egger’s test [31, 32], with *p* < 0.05 considered statistically significant.

## Results

### Literature search

**Fig. 1:**
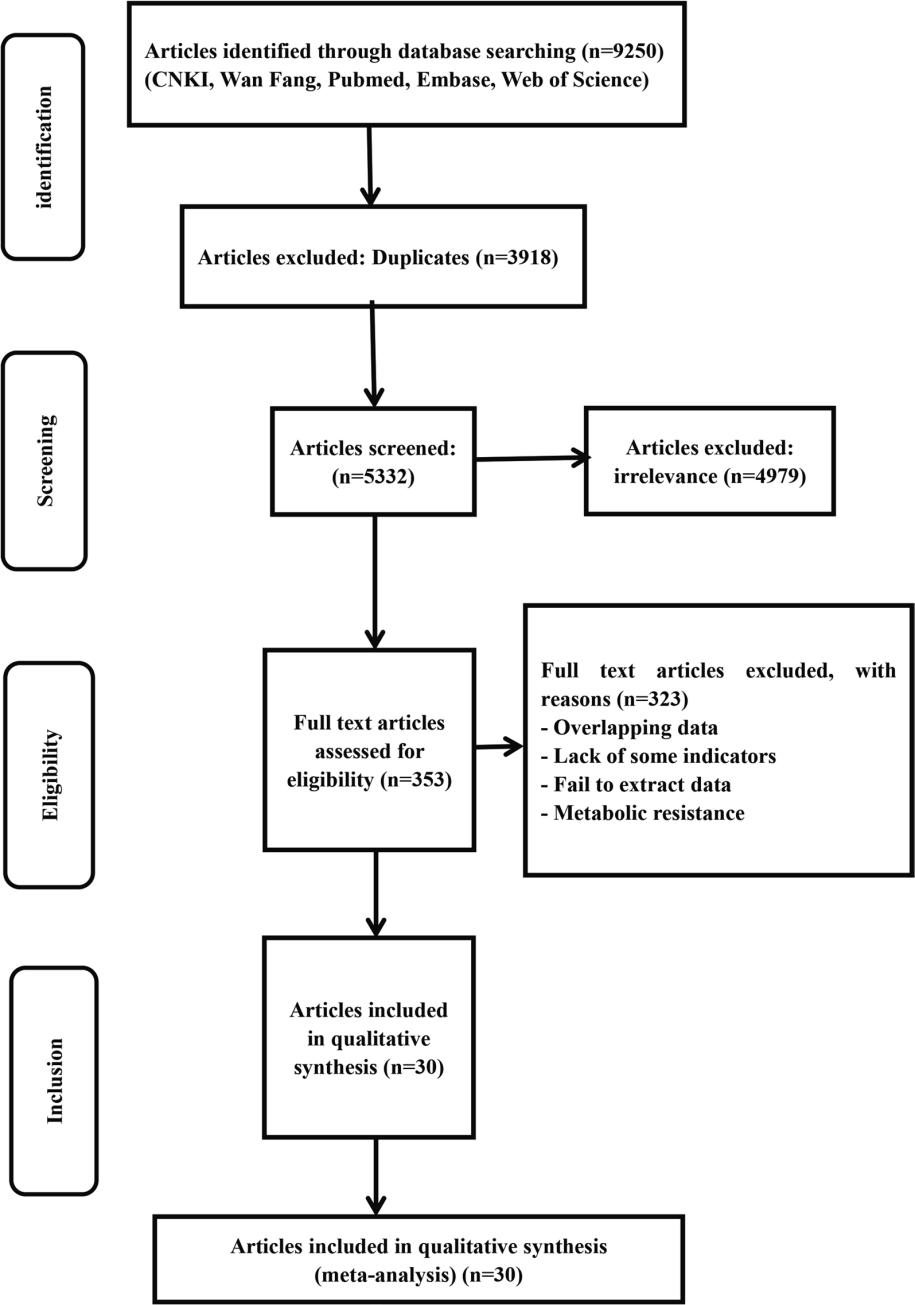
Flow chart of the study selection process in this meta-analysis

A total of 9250 articles were retrieved from the databases. A total of 3918 articles were excluded because they were duplicates, and 4979 irrelevant studies were excluded. Then, 353 articles were evaluated for eligibility, and 323 studies were excluded for the following reasons: inability to extract data; examination of metabolic resistance; overlapping datasets; and missing indicators. After the screening process 30 studies were ultimately included for analysis (Fig. 1).

### Study characteristics

**Fig. 2:**
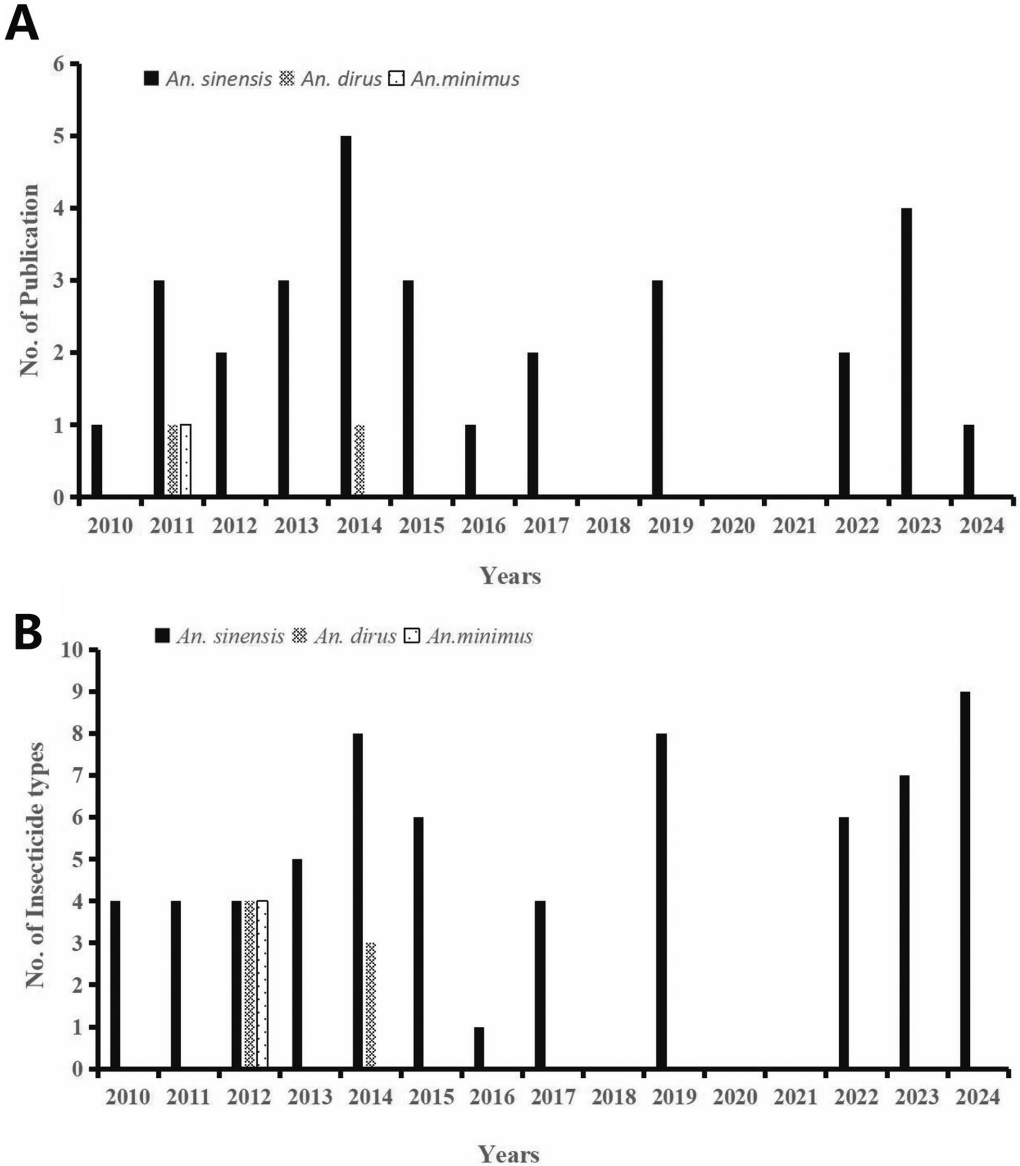
**A** Temporal trends of the insecticide resistance studies of *Anopheles* from 2010 to 2024. **B** Types of insecticides tested on *Anopheles* from 2010 to 2024

The fundamental characteristics and quality of the included studies are presented in Table 1. In all, 30 articles involving 30,065 *An. sinensis* were included. None of the studies were published before 2010; therefore, Fig. 2A presents the temporal trends in insecticide resistance studies of *Anopheles* mosquitoes from 2010–2024. A total of 28 articles focused exclusively on *An. sinensis*; 1 article studied *An. sinensis*, *An. minimus*, and *An. dirus* collectively; and 1 article examined both *An. sinensis* and *An. vagus*. In addition, 11 studies analyzed *kdr* mutations, whereas 7 studies investigated *ace-1* mutation sites. Figure 2B presents the number of insecticide types tested on *Anopheles* from 2010 to 2024. More than six insecticide types were tested on *An. sinensis* after 2022. Over a 15-year period (2010–2024), 30 studies were collected across China, including Henan Province, Jiangsu Province, Liaoning Province, Shandong Province, Hubei Province, Hainan Province, Anhui Province, Yunnan Province, Guizhou Province, Hunan Province, Sichuan Province, Jiangxi Province, Shanghai municipality (a provincial-level administrative division of China), and Chongqing municipality.

**Table 1:** Basic characteristics of the articles included in meta-analysis

First author	Publication year	Sampling area	Study periods	*Anopheles* species	Insecticide types	Insecticide class	Sample size^*^	Number of dead mosquitoes	Language	Quality rating
Hu et al. [33]	2010	Hubei	2007; 2009	*An. sinensis*	DDT, deltamethrin, malathion, and cyfluthrin	Organochlorines, pyrethroids, and organophosphates	2034	365	Chinese	Fair
Wu et al [34]	2011	Anhui	2011	*An. sinensis*	Deltamethrin	Pyrethroids	427	37	Chinese	Fair
Zeng et al. [35]	2011	Hainan	2008–2010	*An. sinensis*, *An. dirus*, and *An. minimus*	DDT, deltamethrin, malathion, and cyfluthrin	Organochlorines, pyrethroids, and organophosphates	1014	806	Chinese	Fair
Li et al. [36]	2011	Jiangsu	NA	*An. sinensis*	DDT, deltamethrin, and malathion	Organochlorines, pyrethroids, and organophosphates	441	296	Chinese	Fair
Liu et al [37]	2012	Henan	2009	*An. sinensis*	DDT, deltamethrin, malathion, and cyfluthrin	Organochlorines, pyrethroids, and organophosphates	1271	1030	Chinese	Fair
Qi et al. [38]	2012	Yunnan and Henan	2010	*An. sinensis*	Deltamethrin	Pyrethroids	496	268	Chinese	Fair
Li et al. [39]	2013	Liaoning	2011–2012	*An. sinensis*	DDT, deltamethrin, propoxur, beta-cypermethrin, and permethrin	Organochlorines, pyrethroids, and carbamates	2497	1776	Chinese	Fair
Fu et al. [40]	2013	Yunnan, Chongqing, Hubei, and Hunan	2014–2015	*An. sinensis*	Deltamethrin	Pyrethroids	626	141	Chinese	Good
Wang et al. [41]	2013	Hubei, Henan, Hunan, Yunnan, Jiangsu, Jiangxi, Sichuan, Shanghai, and Hunan	2010	*An. sinensis*	DDT and deltamethrin	Organochlorines and pyrethroids	1406	445	English	Fair
Chang et al. [42]	2014	Yunnan and Anhui	2012	*An. sinensis*	DDT, deltamethrin, permethrin, malathion, and bendiocarb	Organochlorines, pyrethroids, organophosphates, and carbamates	1194	368	English	Good
Che et al. [43]	2014	Liaoning	NA	*An. sinensis*	DDT, deltamethrin, neta-cypermethrin, cyfluthrin, and propoxur	Organochlorines, pyrethroids, and carbamates	500	466	Chinese	Fair
Dai et al. [44]	2014	Shandong	NA	*An. sinensis*	DDT, deltamethrin, malathion, and cyfluthrin	Organochlorines, pyrethroids, and organophosphates	1275	713	Chinese	Fair
Zhu et al. [45]	2014	Jiangsu	2011	*An. sinensis*	Deltamethrin	Pyrethroids	514	30	Chinese	Good
Qin et al. [46]	2014	Hainan	2012	*An. sinensis* and *An. vagus*	DDT, deltamethrin, and malathion	Organochlorines, pyrethroids, and organophosphates	1468	1251	English	Good
Liu et al. [47]	2015	Yunnan and Sichuan	2014	*An. sinensis*	DDT, deltamethrin, beta-cypermethrin, and cyfluthrin	Organochlorines and pyrethroids	601	228	Chinese	Good
Fan et al. [48]	2015	Shanghai and Hunan	2010–2012	*An. sinensis*	Deltamethrin, beta-cypermethrin, permethrin, fenobucarb, and fenitrothion	Pyrethroids, organophosphates, and carbamates	2041	1151	Chinese	Fair
Dai et al. [49]	2015	Shandong	2003–2012	*An. sinensis*	DDT, deltamethrin, malathion, and cyfluthrin	Organochlorines, pyrethroids, and organophosphates	4370	2363	English	Good
Sun et al. [50]	2016	Yunnan, Henan, and Jiangsu	2014–2015	*An. sinensis*	Deltamethrin	Pyrethroids	472	262	Chinese	Good
Sun et al. [51]	2017	Hainan	2011–2014	*An. sinensis*	DDT, deltamethrin, and malathion	Organochlorines, pyrethroids, and organophosphates	2436	1301	English	Fair
Xu et al. [52]	2017	Jiangxi	NA	*An. sinensis*	Cyfluthrin	Pyrethroids	111	66	Chinese	Fair
Chen et al. [53]	2019	Zhejiang	2015	*An. sinensis*	DDT, deltamethrin, and malathion	Organochlorines, pyrethroids, and organophosphates	676	NA	English	Fair
Fang et al. [54]	2019	Shanghai	2016–2017	*An. sinensis*	Deltamethrin, cyfluthrin, α-cypermethrin, and fenitrothion	Pyrethroids and organophosphates	1032	684	English	Good
Liang et al. [55]	2019	GuiZhou	2017–2018	*An. sinensis*	Deltamethrin, beta-cypermethrin, and permethrin	Pyrethroids	944	568	Chinese	Good
Wei et al. [56]	2022	Shanghai	2021	*An. sinensis*	Deltamethrin, fenitrothion, and lambda-cyhalothrin	Pyrethroids and organophosphates	959	785	Chinese	Fair
Lin et al. [57]	2022	Yunnan	2020	*An. sinensis*	Deltamethrin, malathion, lambda-cyhalothrin, beta-cypermethrin, and propoxur	Pyrethroids, organophosphates, and carbamates	852	487	Chinese	Fair
Zhou et al. [58]	2023	Yunnan	2020	*An. sinensis*	Beta-cypermethrin, lambda-cyhalothrin, deltamethrin, propoxur, malathion, and fenitrothion	Pyrethroids, organophosphates, and carbamates	420	236	Chinese	Fair
Wan et al. [59]	2023	Hubei	2019, 2021	*An. sinensis*	Deltamethrin, malathion, and beta-cyfluthrin	Pyrethroids and organophosphates	776	277	Chinese	Fair
He et al. [60]	2023	Henan	2021	*An. sinensis*	Deltamethrin, propoxur, beta-cyfluthrin, and malathion	Pyrethroids, organophosphates, and carbamates	1334	737	English	Good
Shi et al. [61]	2023	Guizhou	2018–2020	*An. sinensis*	DDT, deltamethrin, and malathion	Organochlorines, pyrethroids, and organophosphates	1090	635	Chinese	Fair
Cao et al. [62]	2024	Yunnan	2023	*An. sinensis*	Deltamethrin, beta-cyfluthrin, lambda-cyhalothrin, malathion, permethrin, fenitrothion, propoxur, bendiocarb, and fipronil	Pyrethroids, organophosphates, carbamates, and phenylpyrazole	2224	1250	Chinese	Fair

*NA* not available, *DDT* dichlorodiphenyltrichloroethane

^*^Means excluding control

### Insecticide types and concentrations

The mortality rate when using the WHO discriminating concentration of around 0.05% deltamethrin was investigated in 27 articles, that of around 0.75% permethrin was examined in 4 articles, that of around 4% dichlorodiphenyltrichloroethane (DDT) was examined in 14 articles, that of around 5% malathion was examined in 14 articles, that of around 0.15% cyfluthrin was examined in 8 articles, that of around 0.1% propoxur was examined in 6 articles, that of around 1% fenitrothion was examined in 5 articles, that of around 0.15% beta-cyfluthrin was examined in 3 articles, that of around 0.05% beta-cypermethrin was examined in 6 articles, and that of around 0.05% lambda-cyhalothrin was examined in 3 articles.

### Insecticide resistance profile among *Anopheles* mosquitoes

**Fig. 3:**
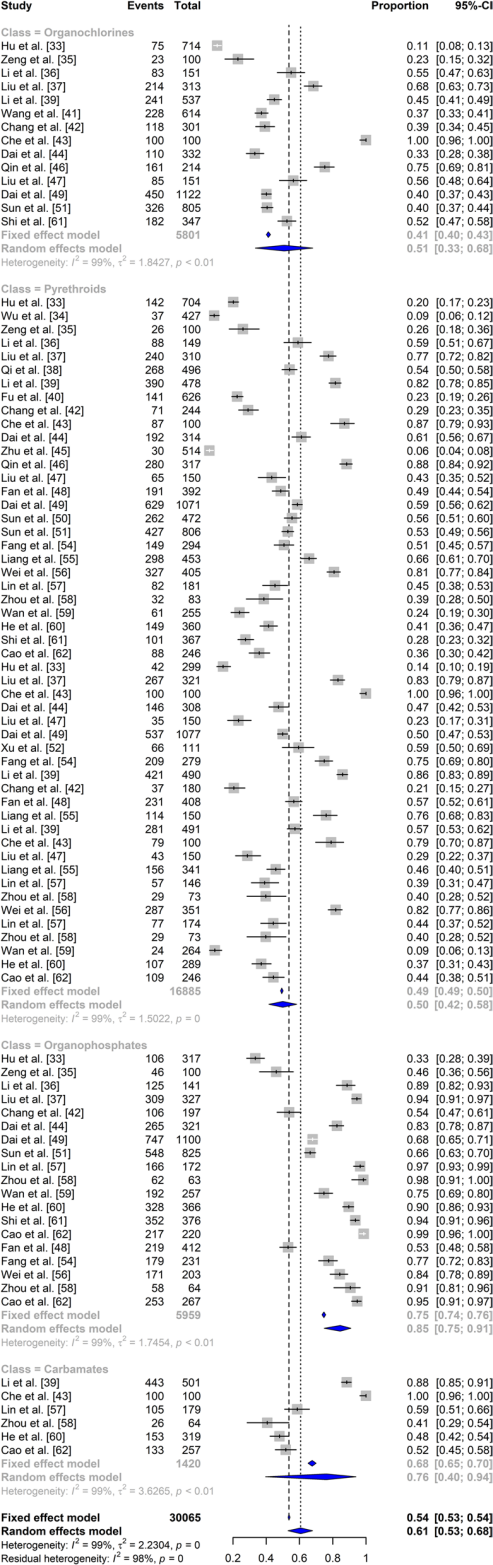
Forest plot of the mortality rates for *An. sinensis* according to the classification of insecticides

The pooled mortality rate for insecticide resistance was 61% [95% confidence interval (CI): 53–68]. Specifically, for organophosphates, carbamates, pyrethroids, and organochlorines, the pooled mortality rates among *An. sinensis* were 85% (95% CI: 75–91), 76% (95% CI: 40–94), 50% (95% CI: 42–58), and 51% (95% CI: 33–68), respectively (Fig. 3). Comprehensive information of the ten insecticides on *An. sinensis* are presented in Additional file 2: Table S2. The overall mortality rates were 49% for DDT, 47% for deltamethrin, 81% for malathion, 69% for propoxur, 61% for permethrin, 28% for beta-cyfluthrin, 82% for fenitrothion, 48% for beta-cypermethrin, 59% for cyfluthrin, and 56% for lambda-cyhalothrin. These results demonstrated that *An. sinensis* mosquitoes were resistant to organophosphate (malathion and fenitrothion) and pyrethroid (beta-cyfluthrin and deltamethrin) insecticides. A subgroup analysis was subsequently performed on that basis of location (province), and the results are shown in Additional file 3: Table S3. Due to the limited number of studies that examined *An. vagus*, *An. minimus*, and *An. dirus*, we could not perform comparative analysis on the other *Anopheles* mosquitoes.

### Frequency of target-site insensitivity resistance mutations

**Fig. 4:**
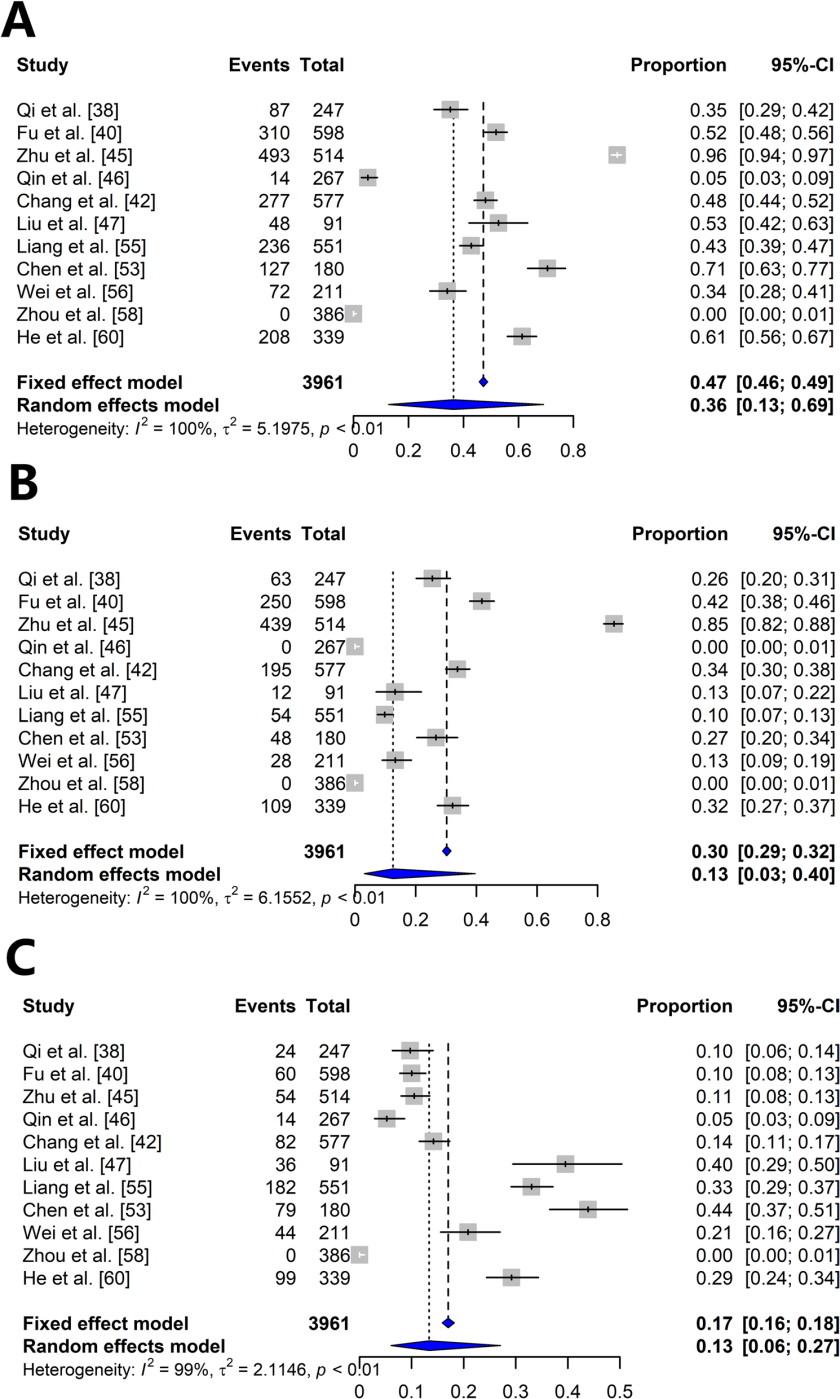
Forest plots based on the random effects model in the meta-analysis. **A** The frequency of *kdr*. **B** The frequency of homozygous resistance to *kdr*. **C** The frequency of heterozygous resistance to *kdr*

**Fig. 5:**
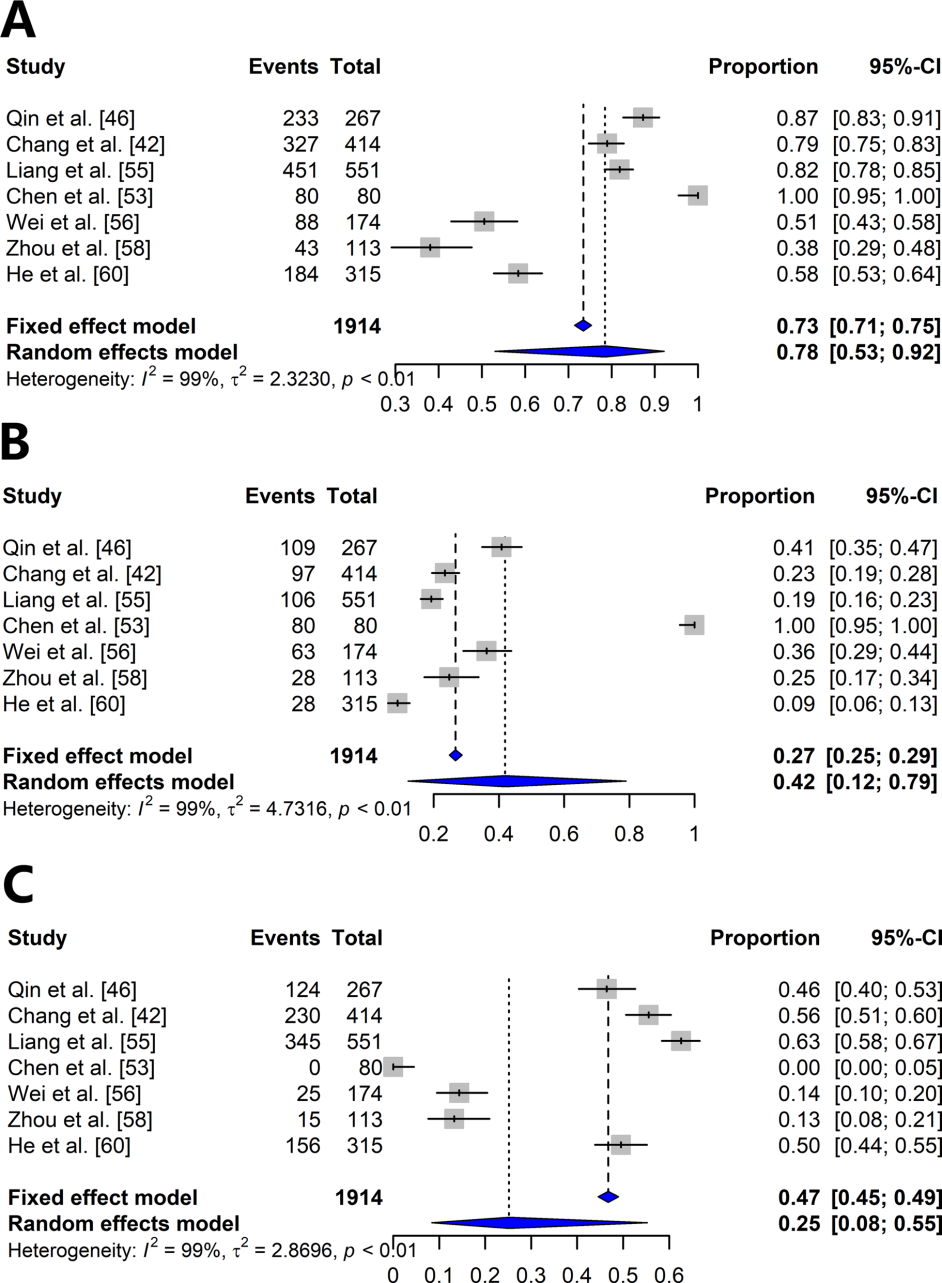
Forest plots based on the random effects model in the meta-analysis. **A** The frequency of *ace-1*. **B** The frequency of homozygous resistance to *ace-1*. **C** The frequency of heterozygous resistance to *ace-1*

The frequency of *kdr* in *An. sinensis* was 36% (95% CI: 13–69), whereas the frequency of *ace-1* resistance was 78% (95% CI: 53–92). Based on *kdr* genotype analysis, the frequency of homozygous resistance (leucine/leucine, phenylalanine/phenylalanine, cysteine/cysteine) was 13%, whereas the frequency of heterozygous resistance (leucine/phenylalanine, cysteine/phenylalanine, tryptophan/phenylalanine, tryptophan/leucine, phenylalanine/leucine, leucine/cysteine, leucine/serine, phenylalanine/tryptophan, cysteine/leucine, phenylalanine/cysteine) was 13%. For *ace-1* mutations, the frequency of homozygous resistance (glycine/glycine and serine/serine) was 42%, whereas the frequency of heterozygous resistance (glycine/serine and serine/glycine) was 25% (Figs. 4, 5).

Two of the incorporated studies [53, 60] performed odds ratio (OR) analyses linking resistance phenotypes to target-site genotypes, which revealed that the frequencies of the *L1014F* *kdr* allele in both deltamethrin (beta-cyfluthrin)- and DDT-resistant mosquitoes were significantly greater than those in sensitive mosquitoes. Significantly greater *G119S* *ace-1* allele frequencies were detected in propoxur- and malathion-resistant mosquitoes than in propoxur- and malathion-sensitive mosquitoes.

Subgroup analysis was performed on the basis of the classification of insecticides. The frequency of the homozygous *kdr* genotype was 10% (pyrethroids 13% and organochlorines 6%), whereas the frequency of the heterozygous genotype was 12% (pyrethroids 10% and organochlorines 18%). The frequency of the homozygous *ace-1* genotype was 42% (organophosphates 72% and carbamates 19%), whereas the frequency of the heterozygous genotype was 48% (organophosphates 46% and carbamates 50%) (Additional file 4: Fig. S1A–C and Additional file 5: Fig. S2A–C).

### Sensitivity analysis and publication bias

A sensitivity analysis was performed, and the results revealed little change in the data (Additional file 6: Table S4). The insecticide mortality rate remained stable and had no significant effect on the pooled results.

Egger’s test and Begg’s test were conducted to evaluate publication bias. The results revealed that the *t* value for Egger’s test was −1.70 (*P* = 0.093), and the *z* value for Begg’s test was 1.77 (*P* = 0.077). When the overall frequency of *kdr* was examined, the *t* value for Egger’s test was −1.33 (*P* = 0.219), and the *z* value for Begg’s test was 0.36 (*P* = 0.721). When the overall frequency of *ace-1* resistance was examined, the *t* value for Egger’s test was −4.70 (*P* = 0.009), and the *z* value for Begg’s test was 1.88 (*P* = 0.060).

## Discussion

China achieved full discontinuation of DDT in 2009, and provisions were made for its potential deployment in vector-borne disease emergencies under the Stockholm Convention Framework [63]. The DDT-associated mortality rate for *An. sinensis* was 49% in our study, which is consistent with findings for other *Anopheles* malaria vectors in Iran (*An. stephensi*), Lao People's Democratic Republic (PDR) (*An. umbrosus*), and Turkey (*An. sacharovi*) [64–66]. *Aedes albopictus* also displayed high resistance to DDT (34%) in China [67]. These observations suggest that other insecticides can be used as alternatives to DDT. Pyrethroids are predominantly utilized as insecticides in IRSs and ITNs, safeguarding humans against indoor mosquito bites. Subgroup analysis (location) revealed pyrethroid resistance in *An. sinensis* populations across all 14 surveyed provinces (municipalities). We recommend (i) the deployment of piperonyl butoxide (PBO), (ii) the use of long-lasting insecticidal nets (LLINs) or dual LLINs (impregnated with both pyrethroid and nonpyrethroid insecticides) and (iii) the application of new chemical insecticides for IRSs. According to the WHO, two novel insecticides, neonicotinoid and pyrrole, were prequalified for IRSs against *Anopheles* [8, 9, 68]. A previous study reported that mosquito resistance to pyrethroid and organochlorine (DDT) insecticides is attributed mainly to point mutations in the L1014 locus of the gene encoding a voltage-gated sodium channel, leading to the decreased binding capacity of insecticides to their target sites [69]. In our study, 36% of the *An. sinensis* populations carried *kdr* mutations, conferring resistance to both organochlorines and pyrethroids. This resistance pattern likely results from sustained selection pressure due to extensive historical use of organochlorines and current pyrethroid-based vector control, thus compromising intervention effectiveness [70].

*Anopheles sinensis* is resistant to organophosphates, with mortality rates of 81% for malathion and 82% for fenitrothion according to WHO bioassays. Propoxur has reached a resistance level (69% mortality rate) in Chinese populations. The results for organophosphate insecticides are similar to those of previously reported studies [65, 71], which may be related to the lower use of malathion or fenitrothion in recent decades [72]. Given the resistance of mosquitoes to organophosphate insecticides, alternative insecticides can be used to prolong the development of resistance to malathion and fenitrothion. Furthermore, possible resistance to malathion and fenitrothion was observed in Yunnan and Henan, and susceptibility to propoxur was observed in Liaoning. An amino acid substitution from glycine to serine in the acetylcholinesterase binding pocket, resulting from a codon 119 mutation in the *ace-1* gene, mediates resistance to both organophosphates and carbamates [73]. Of *An. sinensis* populations, 78% carried *ace-1* mutations, probably leading into reduced susceptibility to organophosphates (83% mortality rate) and carbamates (64% mortality rate). Genotype analysis revealed that the rates of homozygous and heterozygous resistance to *ace-1* were 42% and 29%, respectively. These observations indicate that metabolic resistance also plays a role in the resistance of *An. sinensis* to insecticides. The linkage between resistance phenotypes and target-site genotypes suggests that elevated allele frequencies of mutations (e.g., *kdr* *L1014F* or *ace-1* *G119S*) can predict higher resistance levels in mosquito populations to some extent [53, 60]. Elevated nonspecific esterase activity in mosquitoes mediates resistance to organophosphate, carbamate, and pyrethroid insecticides through enhanced detoxification capacity [74, 75]. Our study revealed increased resistance to the chemical insecticides used for mosquito control. Recent research has demonstrated that host-associated symbiotic microorganisms, particularly *Wolbachia*-based biocontrol systems, offer a promising alternative strategy for disrupting vector-borne disease transmission [76, 77].

Our meta-analysis had several limitations. Firstly, significant heterogeneity brought into question the suitability of performing this meta-analysis. Secondly, we could not analyze some *Anopheles* in these studies due to a lack of data for *An. minimus*, *An. vagus*, and *An. dirus*. Thirdly, the review and analysis did not take into consideration involvement of metabolic and behavioral mechanisms of insecticide resistance.

## Conclusions

In conclusion, an analysis of the literature revealed resistance to carbamate, organophosphate, pyrethroid, and organochlorine insecticides among *An. sinensis*. The associations between resistance phenotypes and *kdr* genotypes suggest that *kdr* mutations likely contribute to pyrethroid and organochlorine resistance mechanisms. Despite the 78% frequency of *ace-1* mutations, the high mortality rate suggests that *ace-1* mutations may not be the dominant resistance mechanism in this vector. Further studies are needed to conduct comprehensive research on resistance mechanisms from the perspectives of enzymology and proteomics. Several strategies are needed, including management plans for selecting insecticides, routine monitoring, and deployment of novel vector control tools.

## Supplementary Information

Additional file 1: Table S1. PRISMA checklist

Additional file 2: Table S2. The mortality rates associated with insecticide exposure in *Anopheles* mosquitoes

Additional file 3: Table S3. The mortality rates associated with insecticide exposure in *Anopheles* mosquitoes. according to location

Additional file 4: Fig. S1. Forest plots based on the random effects model in the meta-analysis according to the classification of insecticides. **A** The frequency of *kdr*. **B** The frequency of homozygous resistance to *kdr*. **C** The frequency of heterozygous resistance to *kdr*

Additional file 5: Fig. S2. Forest plots based on the random effects model in the meta-analysis according to the classification of insecticides. **A** The frequency of ace-1. **B** The frequency of homozygous resistance to *ace-1*. **C** The frequency of heterozygous resistance to *ace-1*

Additional file 6: Table S4. Sensitivity analysis in this review

## Data Availability

The data supporting the findings of the study must be available within the article and/or its supplementary materials, or deposited in a publicly available database.

## Abbreviations

WHO: World Health Organization
IRSs: Indoor residual sprayings
ITNs: Insecticide-treated mosquito nets
LLINs: Long-lasting insecticidal nets
SC: Sodium ion channels
GABA: γ-Aminobutyric acid
ESTs: Esterases
GSTs: Glutathione S-transferases
P450s: P450 monooxygenase system
*kdr*: Knockdown resistance
*ace-1*: Acetylcholinesterase-1
PRISMA: Preferred Reporting Items for Systematic Reviews and Meta-analyses
PROSPERO: International Prospective Register of Systematic Review
CNKI: Chinese National Knowledge Infrastructure databases
NA: Not available
CI: Confidence interval
CR: Confirmed resistance
PR: Possible resistance
S: Susceptible
OR: Odds ratio
DDT: Dichlorodiphenyltrichloroethane
PBO: Piperonyl butoxide
