# Supplementary material for: Insecticide resistance and the role of target-site insensitivity mutations among malaria vectors in China: A systematic review and meta-analysis
Source: Parasit Vectors. 2025 Sep 24;18:374. doi: 10.1186/s13071-025-07020-6 (PMC12462112; doi:10.1186/s13071-025-07020-6)
Supplement: Supplementary file 2 — Additional file 2: Table S2. The mortality rates associated with insecticide exposure in Anopheles mosquitoes [file 13071_2025_7020_MOESM2_ESM.docx]

**Table S2** The mortality rates associated with insecticide exposure in *Anopheles* mosquitoes.

| Types | Included studies | Number of dead mosquitoes | Meta-analysis, pooled rate (95% CI) | Heterogeneity, *P*-value (*I*-squared) |
| --- | --- | --- | --- | --- |
| DDT | 14 | 5801 | 0.49 (0.35-0.64) | <0.01 (98.6%) |
| Deltamethrin | 27 | 10314 | 0.47 (0.38-0.57) | <0.01 (99.0%) |
| Malathion | 14 | 4782 | 0.81 (0.69-0.90) | <0.01 (98.6%) |
| Propoxur | 6 | 1420 | 0.69 (0.44-0.90) | <0.01 (98.6%) |
| Permethrin | 4 | 1228 | 0.61 (0.31-0.87) | <0.01 (99.0%) |
| Beta-cyfluthrin | 3 | 799 | 0.28 (0.09-0.53) | <0.01 (98.1%) |
| Fenitrothion | 5 | 1177 | 0.82 (0.66-0.93) | <0.01 (98.0%) |
| Beta-cypermethrin | 6 | 1301 | 0.48 (0.34-0.63) | <0.01 (94.5%) |
| Cyfluthrin | 8 | 2645 | 0.59 (0.34-0.82) | <0.01 (99.0%) |
| Lambda-cyhalothrin | 3 | 598 | 0.56 (0.29-0.82) | <0.01 (98.0%) |
