# Supplementary material for: Insecticide resistance and the role of target-site insensitivity mutations among malaria vectors in China: A systematic review and meta-analysis
Source: Parasit Vectors. 2025 Sep 24;18:374. doi: 10.1186/s13071-025-07020-6 (PMC12462112; doi:10.1186/s13071-025-07020-6)
Supplement: Supplementary file 3 — Additional file 3: Table S3. The mortality rates associated with insecticide exposure in Anopheles mosquitoes. according to location [file 13071_2025_7020_MOESM3_ESM.docx]

**Table S3** The mortality rates associated with insecticide exposure in *Anopheles* mosquitoes according to location.

| Types | Included provinces | Number of mosquitoes | Meta-analysis, pooled rate (95% CI) | Heterogeneity, *P*-value (*I*-squared) |
| --- | --- | --- | --- | --- |
| DDT | 13 | 5801 | 0.45 (0.32-0.59) | <0.01 (96.6%) |
| Deltamethrin | 13 | 10314 | 0.44 (0.35-0.53) | <0.01 (97.9%) |
| Malathion | 8 | 4782 | 0.83 (0.70-0.92) | <0.01 (97.5%) |
| Propoxur | 3 | 1420 | 0.76 (0.40-0.94) | <0.01 (97.1%) |
| Permethrin | 6 | 1228 | 0.52 (0.28-0.75) | <0.01 (97.6%) |
| Beta-cyfluthrin | 3 | 799 | 0.27 (0.11-0.50) | <0.01 (97.2%) |
| Fenitrothion | 3 | 1177 | 0.79 (0.59-0.90) | <0.01 (96.7%) |
| Beta-cypermethrin | 4 | 1301 | 0.46 (0.33-0.59) | <0.01 (92.2%) |
| Cyfluthrin | 8 | 2645 | 0.59 (0.29-0.84) | <0.01 (97.6%) |
| Lambda-cyhalothrin | 3 | 598 | 0.57 (0.33-0.78) | <0.01 (96.0%) |
