# Supplementary material for: Insecticide resistance and the role of target-site insensitivity mutations among malaria vectors in China: A systematic review and meta-analysis
Source: Parasit Vectors. 2025 Sep 24;18:374. doi: 10.1186/s13071-025-07020-6 (PMC12462112; doi:10.1186/s13071-025-07020-6)
Supplement: Supplementary file 4 — Additional file 4: Fig. S1. Forest plots based on the random effects model in the meta-analysis according to the classification of insecticides. A The frequency of kdr. B The frequency of homozygous resistance to kdr. C The frequency of heterozygous resistance to kdr [file 13071_2025_7020_MOESM4_ESM.docx]

**Table S4** Sensitivity analysis in this review.

| Types | Included studies | Meta-analysis, pooled rate (95% CI) | Heterogeneity, P-value (*I*-squared) | Sensitive analysis (remove single case data set) | Heterogeneity, *P*-value (*I*-squared) |
| --- | --- | --- | --- | --- | --- |
| DDT | 14 | 0.49 (0.35-0.64) | <0.01 (98.6%) | 0.43 (0.33-0.57) | <0.01 (99.5%) |
| Deltamethrin | 27 | 0.47 (0.38-0.57) | <0.01 (99.0%) | 0.41 (0.32-0.52) | <0.01 (98.6%) |
| Malathion | 14 | 0.81 (0.69-0.90) | <0.01 (98.6%) | 0.75 (0.63-0.88) | <0.01 (98.4%) |
| Propoxur | 6 | 0.69 (0.44-0.90) | <0.01 (98.6%) | 0.62(0.47-0.82) | <0.01 (98.8%) |
| Permethrin | 4 | 0.61 (0.31-0.87) | <0.01 (99.0%) | 0.53 (0.29-0.99) | <0.01 (98.2%) |
| Beta-cyfluthrin | 3 | 0.28 (0.09-0.53) | <0.01 (98.1%) | 0.25 (0.09-0.66) | <0.01 (96.6%) |
| Fenitrothion | 5 | 0.82 (0.66-0.93) | <0.01 (98.0%) | 0.79 (0.64-0.96) | <0.01 (97.5%) |
| Beta-cypermethrin | 6 | 0.48 (0.34-0.63) | <0.01 (94.5%) | 0.46 (0.35-0.62) | <0.01 (95.1%) |
| Cyfluthrin | 7* | 0.59 (0.34-0.82) | <0.01 (99.0%) | 0.49 (0.31-0.77) | <0.01 (99.3%) |
| Lambda-cyhalothrin | 3 | 0.56 (0.29-0.82) | <0.01 (98.0%) | 0.53 (0.34-0.83) | <0.01 (97.1%) |
| *kdr* | 11 | 0.43 (0.23-0.64) | <0.01 (99.5%) | 0.35 (0.20-0.63) | <0.01 (99.1%) |
| *ace-1* | 7 | 0.74 (0.54-0.90) | <0.01 (97.8%) | 0.68 (0.53-0.87) | <0.01 (98.4%) |

Note：*means excluding study with a probability of 1.
