# Supplementary figures and images for: Insecticide resistance and the role of target-site insensitivity mutations among malaria vectors in China: A systematic review and meta-analysis
Source: Parasit Vectors. 2025 Sep 24;18:374. doi: 10.1186/s13071-025-07020-6 (PMC12462112; doi:10.1186/s13071-025-07020-6)

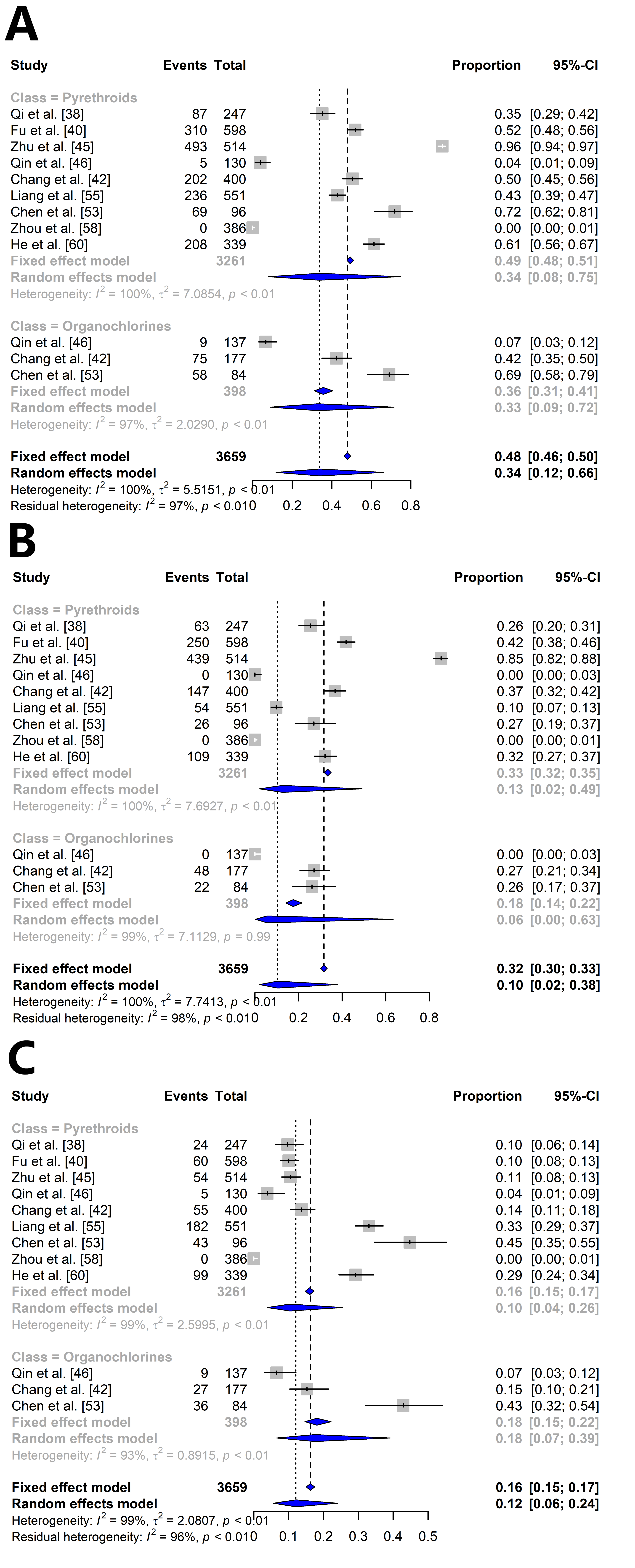

Supplement: Supplementary file 5 — Additional file 5: Fig. S2. Forest plots based on the random effects model in the meta-analysis according to the classification of insecticides. A The frequency of ace-1. B The frequency of homozygous resistance to ace-1. C The frequency of heterozygous resistance to ace-1 [file 13071_2025_7020_MOESM5_ESM.tif]

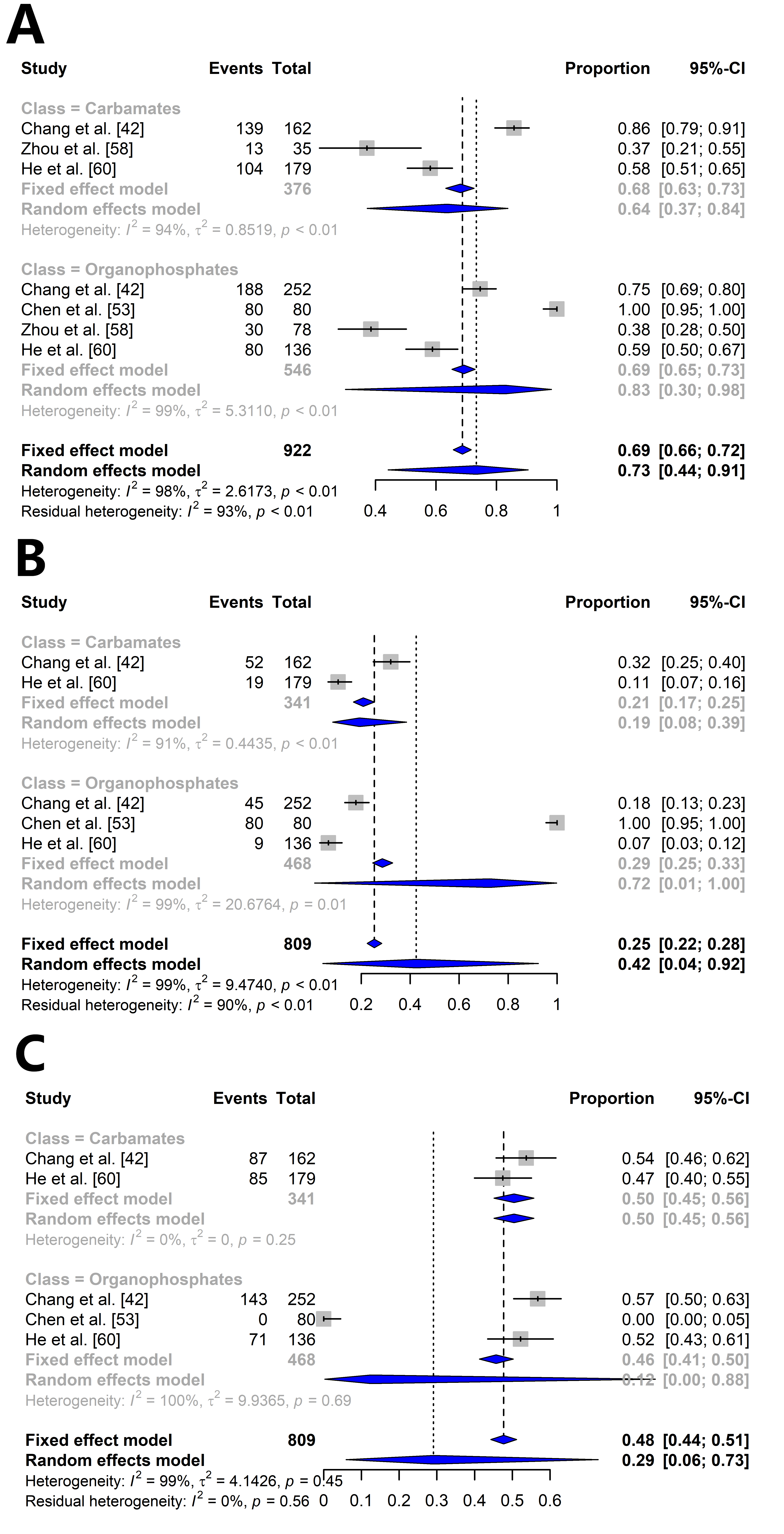

Supplement: Supplementary file 6 — Additional file 6: Table S4. Sensitivity analysis in this review [file 13071_2025_7020_MOESM6_ESM.tif]
